# *Philodromus uljin* sp. nov., a new running crab spider (Araneae, Philodromidae) from South Korea

**DOI:** 10.3897/BDJ.14.e178451

**Published:** 2026-02-11

**Authors:** Chang Moon Jang, Sue Yeon Lee, Seung Tae Kim

**Affiliations:** 1 Diversity Conservation Research Department, Nakdonggang National Institute of Biological Resources, Ministry of Environment, Sangju 37242, Republic of Korea Diversity Conservation Research Department, Nakdonggang National Institute of Biological Resources, Ministry of Environment Sangju 37242 Republic of Korea; 2 R&D Center, Cellcuratio Co., Ltd., Daejeon 34054, Republic of Korea R&D Center, Cellcuratio Co., Ltd. Daejeon 34054 Republic of Korea; 3 Life and Environment Research Institute, Konkuk University, Seoul, Republic of Korea Life and Environment Research Institute, Konkuk University Seoul Republic of Korea

**Keywords:** biodiversity, morphology, Philodromid spider, species description, taxonomy

## Abstract

**Background:**

Philodromidae Thorell, 1869 is a spider family comprising 531 species in 31 genera distributed worldwide. Amongst these genera, *Philodromus* Walckenaer, 1826 is the most diverse with 211 described species.

**New information:**

A new running crab spider, *Philodromus uljin* **sp. nov.**, is described from Uljin-gun, Gyeongsangbuk-do, South Korea. This study provides a diagnosis, morphological description and photographs of the habitat, live habitus and genital organs of both males and females.

## Introduction

**Figure 1.**
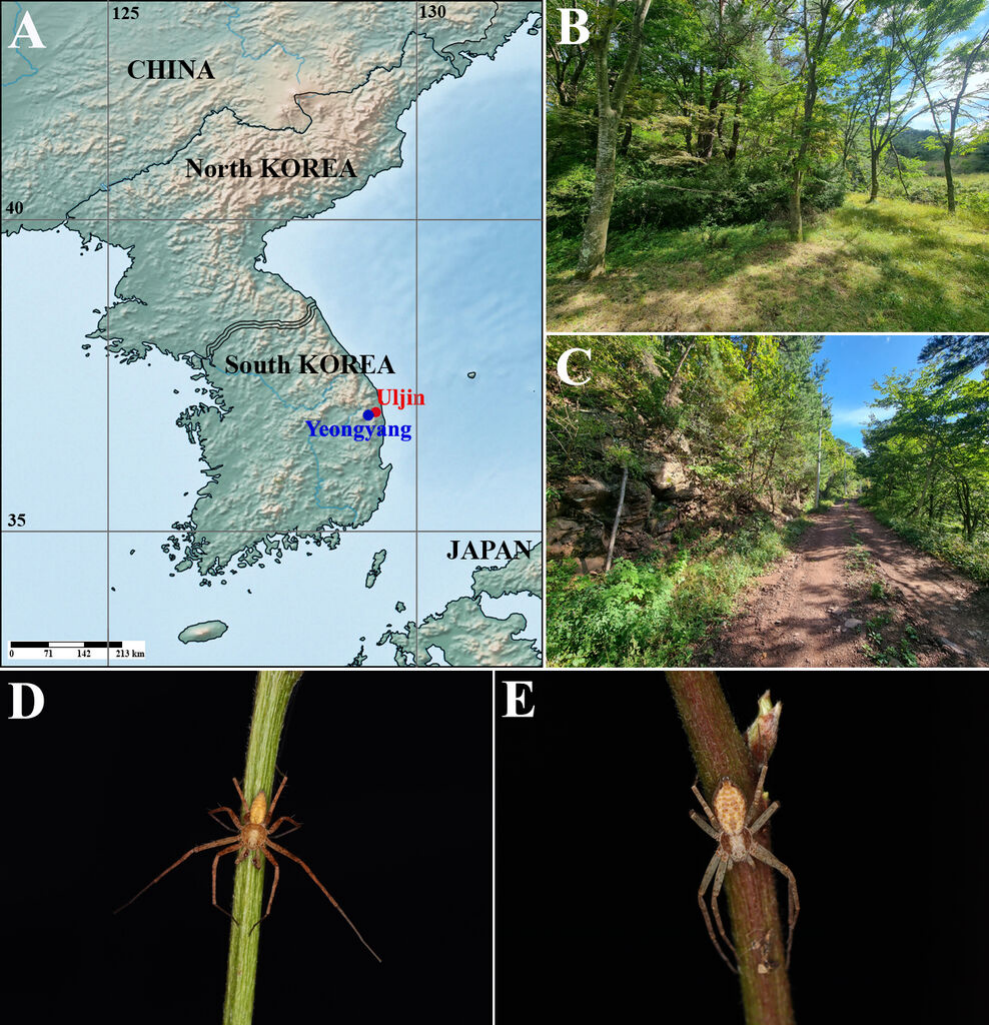
*Philodromus uljin* sp. nov. **A** Distribution map; **B** Habitat (Uljin-gun, Gyeongsangbuk-do); **C** Habitat (Yeongyang-gun, Gyeongsangbuk-do); **D** Male, live habitus; **E** Female, live habitus.

The spiders of the genus *Philodromus* Walckenaer, 1826 are commonly found in grasses, shrubs, tree trunks, low herbage and undergrowth (Schick 1965, Levy 1977, Dondale and Redner 1978). Of the 211 species classified within the genus worldwide, nine species have been recorded in Korea: *P. aureolus* (Clerck, 1757), *P. auricomus* L. Koch, 1878, *P. cespitum* (Walckenaer, 1802), *P. emarginatus* (Schrank, 1803), *P. margaritatus* (Clerck, 1757), *P. paiki* Jang, Lee, Yoo & Kim, 2024, *P. rufus* Walckenaer, 1826, *P. spinitarsis* Simon, 1895 and *P. subaureolus* Bösenberg & Strand, 1906 (World Spider Catalog 2025). In recent years, taxonomic research on this genus has been relatively limited. Although *Philodromus* is well known for its high species diversity, the genus remains inadequately studied and its diversity is still poorly understood (Zhang et al. 2025). In recent taxonomic studies of the genus *Philodromus* in South Korea, *P. pseudoexilis* Paik, 1979, previously recorded as an endemic species to the country, was synonymised with *P. rufus* Walckenaer, 1826 (Jang et al. 2024). Furthermore, the species, formerly identified as *P. poecilus* (Thorell, 1872), was determined to have been misidentified and has been newly described as *P. paiki* Jang, Lee, Yoo & Kim, 2023 (Jang et al. 2023). In addition, a comprehensive taxonomic and genetic study has recently been conducted in China on seven species belonging to the genus *Philodromus*, together with the genera *Tibellus* and *Sinodromus*, which inhabit the Guizhou and Hubei Provinces (Zhang et al. 2025). The spider fauna of mountainous mixed forests in Gangwon-do and Gyeongsangbuk-do was intensively surveyed between 2023 and 2025. During the survey, both males and females of *Philodromus uljin* **sp. nov.** were collected from shrubby vegetation layers using a sweeping net (Fig. 1). The present paper provides a taxonomic description of *Philodromus uljin* **sp. nov.**, including its diagnosis, measurements, morphological illustrations, distribution map and photographs of the habitat and live habitus.

## Materials and methods

All specimens were preserved in 98% ethyl alcohol and external morphology was examined under a Leica S8APO (Singapore) stereomicroscope. Images were captured with a Dhyana 400DC zoom digital camera (China) mounted on a Leica S8APO and assembled using Helicon Focus 8.3.7 image stacking software (Khmelik et al. 2006). Measurements of body parts were made with an ocular micrometer and are recorded in millimeter. Internal genitalia of females were removed and treated in 10% potassium hydroxide (KOH) for 2 hours before illustration. Leg measurements are shown as: Total length (femur, patella, tibia, metatarsus, tarsus). Morphological terminology follows established usage in previous studies (Muster and Thaler 2004, Zhang et al. 2025). The type specimens are deposited in the National Institute of Biological Resources (NIBR), Incheon and Konkuk University (KKU), Seoul, Korea. The following abbreviations are used in the descriptions: **ALE** = anterior lateral eye, **AME** = anterior median eye, **PLE** = posterior lateral eye, **PME** = posterior median eye, **ALE-AME** = distance between ALE and AME, **ALE**-**PLE** = distance between ALE and PME, **AME**-**AME** = distance between AMEs, **AME**-**PME** = distance between AME and PME, **PLE**-**PME** = distance between PLE and PME, **PME**-**PME** = distance between PMEs, **AER** = anterior eye row, **PER** = posterior eye row.

## Taxon treatments

### Philodromus uljin sp. nov.

B8E7AE26-442F-5B94-935E-F2C9359EF376

B54E238C-8880-406B-B972-A8F2E24CB982

#### Materials

- **Type status:** Holotype. **Occurrence:** catalogNumber: TTQXIV0000000649; recordedBy: Chang Moon Jang, Sue Yeon Lee & Seung Tae Kim; individualCount: 1; sex: male; lifeStage: adult; occurrenceID: 07FB0366-DFEE-5A0F-9856-91448AC42D7A; **Taxon:** scientificName: *Philodromus uljin*; kingdom: Animalia; phylum: Arthropoda; class: Arachnida; order: Araneae; family: Philodromidae; **Location:** country: South Korea; stateProvince: Gyeongsangbuk-do; municipality: Uljin-gun; locality: Onjeong-myeon, Oeseonmi-ri, Kuju-ryeoung Pass; verbatimElevation: 552 m; verbatimCoordinates: 36°45'32.6"N 129°18'06.6"E; **Identification:** identifiedBy: Seung Tae Kim; **Event:** samplingProtocol: sweeping; eventDate: 19-06-2024; habitat: mixed forest; **Record Level:** type: Holotype; rights: en; institutionID: National Institute of Biological resources (NIBR)
- **Type status:** Paratype. **Occurrence:** catalogNumber: TTQXIV0000000650; recordedBy: Chang Moon Jang, Sue Yeon Lee & Seung Tae Kim; individualCount: 1; sex: female; lifeStage: adult; occurrenceID: C7337C49-08A5-56D4-A0F8-5224B07C92BD; **Taxon:** scientificName: *Philodromus uljin*; kingdom: Animalia; phylum: Arthropoda; class: Arachnida; order: Araneae; family: Philodromidae; **Location:** country: South Korea; stateProvince: Gyeongsangbuk-do; municipality: Uljin-gun; locality: Onjeong-myeon, Oeseonmi-ri, Kuju-ryeoung Pass; verbatimElevation: 552 m; verbatimCoordinates: 36°45'32.6"N 129°18'06.6"E; **Identification:** identifiedBy: Seung Tae Kim; **Event:** samplingProtocol: sweeping; eventDate: 19-06-2024; habitat: mixed forest; **Record Level:** type: Paratype; language: en; institutionCode: National Institute of Biological resources (NIBR)
- **Type status:** Paratype. **Occurrence:** catalogNumber: NIBRIV0000920275; recordedBy: Chang Moon Jang, Sue Yeon Lee & Seung Tae Kim; individualCount: 1; sex: female; lifeStage: adult; occurrenceID: B77EA26E-4069-5E7A-BD50-2823113A6987; **Taxon:** scientificName: *Philodromus uljin*; kingdom: Animalia; phylum: Arthropoda; class: Arachnida; order: Araneae; family: Philodromidae; **Location:** country: South Korea; stateProvince: Gyeongsangbuk-do; municipality: Uljin-gun; locality: Onjeong-myeon, Oeseonmi-ri, Kuju-ryeoung Pass; verbatimElevation: 552 m; verbatimCoordinates: 36°45'32.6"N 129°18'06.6"E; **Identification:** identifiedBy: Seung Tae Kim; **Event:** samplingProtocol: sweeping; eventDate: 19-06-2024; habitat: mixed forest; **Record Level:** language: en; institutionCode: National Institute of Biological resources (NIBR)
- **Type status:** Paratype. **Occurrence:** catalogNumber: KKU-ARA_Phil_P. uljin_20240619_01; recordedBy: Chang Moon Jang, Sue Yeon Lee & Seung Tae Kim; individualCount: 1; sex: female; lifeStage: adult; occurrenceID: 971036D7-173B-5D85-9BB3-8A923F0E9D65; **Taxon:** scientificName: *Philodromus uljin*; kingdom: Animalia; phylum: Arthropoda; class: Arachnida; order: Araneae; family: Philodromidae; **Location:** country: South Korea; stateProvince: Gyeongsangbuk-do; municipality: Uljin-gun; locality: Onjeong-myeon, Oeseonmi-ri, Kuju-ryeoung Pass; verbatimElevation: 552 m; verbatimCoordinateSystem: 36°45'32.6"N 129°18'06.6"E; **Identification:** identifiedBy: Seung Tae Kim; **Event:** samplingProtocol: sweeping; eventDate: 19-06-2024; habitat: mixed forest; **Record Level:** institutionCode: Konkuk University (KKU)
- **Type status:** Paratype. **Occurrence:** catalogNumber: KKU-ARA_Phil_P. uljin_20241005_02; recordedBy: Chang Moon Jang, Sue Yeon Lee & Seung Tae Kim; individualCount: 1; sex: male; lifeStage: adult; occurrenceID: 0F2FB0D6-8A42-5795-96C8-3B558D8E4A04; **Taxon:** scientificName: *Philodromus uljin*; kingdom: Animalia; phylum: Arthropoda; class: Arachnida; order: Araneae; family: Philodromidae; **Location:** country: South Korea; stateProvince: Gyeongsangbuk-do; municipality: Yeongyang-gun; locality: Subi-myeon, Bonsin-ri, Kuju-ryeoung Pass; verbatimElevation: 587 m; verbatimCoordinates: 36°45'32.0"N 129°17'46.6"E; **Identification:** identifiedBy: Seung Tae Kim; **Event:** samplingProtocol: sweeping; eventDate: 05-10-2024; habitat: mixed forest; **Record Level:** institutionCode: Konkuk University (KKU)
- **Type status:** Paratype. **Occurrence:** catalogNumber: KKU-ARA_Phil_P. uljin_20241005_03; recordedBy: Chang Moon Jang, Sue Yeon Lee & Seung Tae Kim; individualCount: 1; sex: female; lifeStage: adult; occurrenceID: B5B81FBD-DAF9-52B1-88D3-C186EC504064; **Taxon:** scientificName: *Philodromus uljin*; kingdom: Animalia; phylum: Arthropoda; class: Arachnida; order: Araneae; family: Philodromidae; **Location:** country: South Korea; stateProvince: Gyeongsangbuk-do; municipality: Yeongyang-gun; locality: Subi-myeon, Bonsin-ri, Kuju-ryeoung Pass; verbatimElevation: 587 m; verbatimCoordinates: 36°45'32.0"N 129°17'46.6"E; **Identification:** identifiedBy: Seung Tae Kim; **Event:** samplingProtocol: sweeping; eventDate: 05-10-2024; habitat: mixed forest; **Record Level:** institutionCode: Konkuk University (KKU)

#### Description

**Figure 2.**
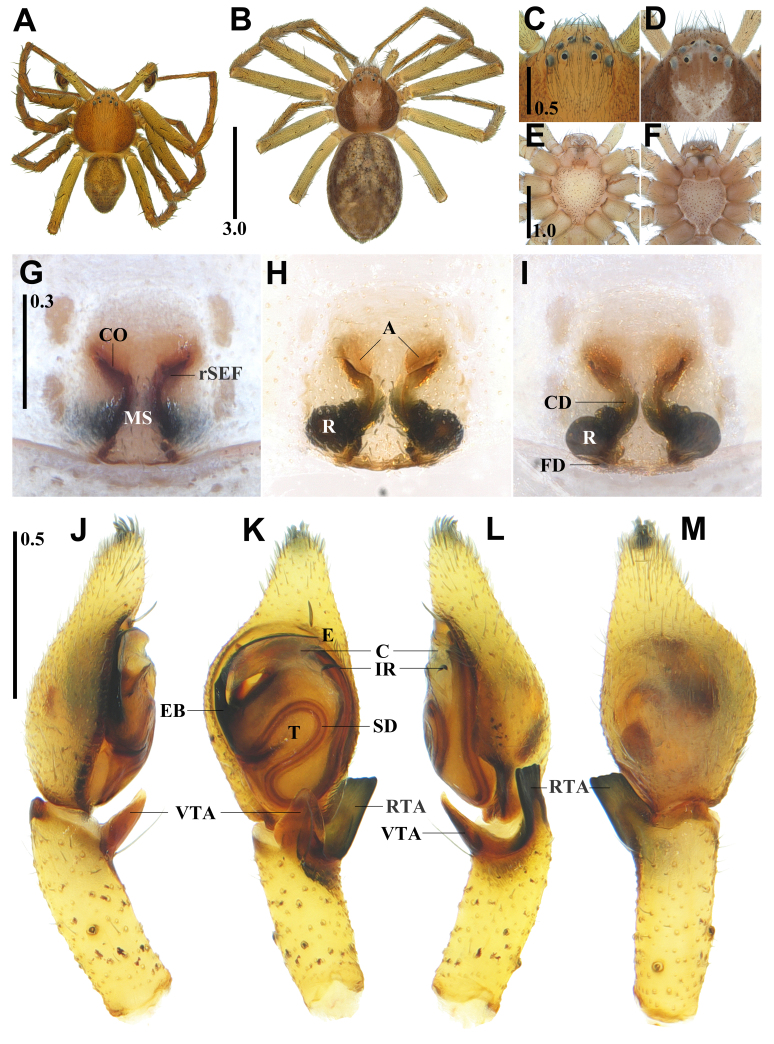
*Philodromus uljin* sp. nov. **A** Male habitus, dorsal view; **B** Female habitus, dorsal view; **C** Male eye area from above; **D** Female eye area from above; **E** Male sternum; **F** Female sternum; **G** Female epigynum, ventral view; **H-I** Internal genitalia, ventral and dorsal views; **J-M** Male palp, prolateral, ventral, retrolateral and dorsal views (C = conductor, CD = copulatory duct, CO = copulatory opening, E = embolus, A = atrium, EB = embolic base, FD = fertilisation duct, IR = intertegular retinaculum, MS = median septum, R = receptaculum, RTA = retrolateral tibial apophysis, rSEF = rim of sclerotised epigynal fold, SD = sperm duct, T = tegulum, VTA = ventral tibial apophysis). Scale bars in mm.

**Male (holotype).** Habitus as in Fig. 2A. Total length 3.95. Carapace 1.99 long / 1.97 wide. Eyes: AER 0.57, PER 0.85, ALE 0.09, AME 0.09, PLE 0.11, PME 0.07, ALE-AME 0.06, ALE-PLE 0.19, AME-AME 0.13, AME-PME 0.16, PLE-PME 0.18, PME-PME 0.29. Chelicera 0.72 long / 0.29 wide. Endite 0.44 long / 0.28 wide. Labium 0.28 long / 0.28 wide. Sternum 1.06 long / 1.06 wide. Legs: I 12.04 (3.11, 1.23, 2.99, 3.00, 1.70), II 14.19 (3.54, 1.31, 3.60, 3.50, 2.25), III 9.77 (2.72, 1.01, 2.25, 2.41, 1.39), IV 10.09 (2.83, 0.96, 2.35, 2.53 1.46). Palp 3.77 (1.48, 0.74, 0.69, - , 0.86). Abdomen 1.88 long / 1.31 wide.

Carapace round and deep orange, longer than wide, thoracic region reddish-brown laterally, clothed densely with short blackish-brown hairs, cervical furrow and fovea indistinct, radial furrow distinct (Fig. 2A). All eyes on eye tubercles, arranged in two rows, anterior eye row recurved and posterior eye row slightly recurved, posterior median eyes smaller than others (Fig. 2A and C). Chelicera pale light pink and strongly developed, with two promarginal teeth. Sternum subcordate, pale light orange, convex, with brown spots scattered, clothed sparsely with short, recumbent hairs, anterior end slightly depressed, posterior tip blunt and slightly protruding between coxae IV (Fig. 2E). Legs pale blackish-brown, clothed densely with short blackish-brown hairs, leg spination: I (femur 0-1-1-2-1-2-3d/0v, tibia 3-2-3d/2-2-2v, metatarsus 3-2-2d/2-2-3v), II (femur 0-1-1-2-1-2-3d/0v, tibia 3-2-3d/2-2-2v, metatarsus 3-2-2d/2-2-3v), III (femur 0-1-1-3-2-3d/0v, tibia 3-2-3d/2-2-2v, metatarsus 3-2-2d/2-2-3v), IV (femur 0-1-1-2-1-2-3d/0v, tibia 3-2-3d/2-2-2v, metatarsus 3-2-2d/2-2-3v), leg formula II-I-IV-III (Fig. 2A). Abdomen ovoid, dusky and pale blackish-brown, clothed densely with blackish-brown hairs, dark anterolaterally, with a blackish-brown lozenge-shaped pattern anteromedially along with two pairs of orange markings, wrinkled posteriorly (Fig. 2A).

Palp (Fig. 2J–M): tegulum elliptical; embolus filiform, embolic base thick and smooth; conductor broad and membranous; intertegular retinaculum small and spine-shaped; ventral tibial apophysis triangular, strongly sclerotised and membranous; retrolateral tibial apophysis rectangular, with an undulated tip, the middle section flat, almost uniform in width.

**Female (paratype).** General appearance similar to male, habitus as in Fig. 2B. Total length 5.51. Carapace: 2.07 long / 2.01 wide. Eyes: AER 0.66, PER 0.97, ALE 0.11, AME 0.09, PLE 0.11, PME 0.08, ALE-AME 0.07, ALE-PLE 0.23, AME-AME 0.15, AME-PME 0.21, PLE-PME 0.20, PME-PME 0.34. Chelicera: 0.79 long / 0.36 wide. Endite: 0.46 long / 0.27 wide. Labium: 0.31 long / 0.30 wide. Sternum: 1.08 long / 1.02 wide. Legs: I 7.05 (1.98, 0.86, 1.64, 1.54, 1.02), II 8.40 (2.41, 0.98, 2.07, 1.84, 1.10), III 6.16 (1.84, 0.77, 1.40, 1.32, 0.82), IV 6.15 (1.88, 0.71, 1.38, 1.34, 0.83). Palp: 2.49 (0.80, 0.44, 0.47, - , 0.79). Abdomen 3.34 long / 2.33 wide. Epigynum 0.30 wide.

Carapace round and pale brown, longer than wide, cephalic region ivory posteriorly, thoracic region tinged with white on anterior two-thirds and dark brown laterally, clothed densely with short blackish-brown hairs, cervical and radial furrows distinct, fovea V-shaped (Fig. 2B). All eyes on eye tubercles, arranged in two rows, anterior eye row recurved and posterior eye row slightly recurved, posterior median eyes smaller than others (Fig. 2B and D). Chelicera pale light pink and strongly developed, with two promarginal teeth. Sternum subcordate, pale light pink, convex, with brown spots scattered, clothed sparsely with short and recumbent hairs, anterior end slightly depressed, posterior tip truncated and slightly protruding between coxae IV (Fig. 2E). Legs pale greenish-brown, clothed densely with short blackish-brown hairs, leg spination: I (femur 0-1-1-2-1-2-3d/0v, tibia 3-2-3d/2-2-2v, metatarsus 3-2-2d/2-2-3v), II (femur 0-1-1-2-1-2-3d/0v, tibia 3-2-3d/2-2-2v, metatarsus 3-2-2d/2-2-3v), III (femur 0-1-1-2-3d/0v, tibia 3-2-3d/2-2-2v, metatarsus 3-2-2d/2-2-3v), IV (femur 0-1-1-3d/0v, tibia 3-2-3d/2-2-2v, metatarsus 3-2-2d/2-2-3v), leg formula II-I-III≈IV (Fig. 2B).

Epigynum (Fig. 2G): epigynal atrium semi-circular and wide; median septum narrow anteriorly and broad posteriorly; anterior rim of sclerotised epigynal fold angularly curved. Internal genitalia (Fig. 2H and I): receptaculum large and globular; globular mound indistinct; copulatory duct distinct, thick, looped and thinner than receptaculum; fertilisation duct almost horizontal.

#### Diagnosis

*Philodromus uljin* sp. nov. is similar to *P. guiyang* Long & Yu, 2002 and *P. subaureolus* Bösenberg & Strand, 1906, in the general shape of the genital organs and overall body appearance, but it can be easily distinguished from both congeners by the following combination of morphological characters. Comparison with *P. guiyang*: Male – embolic base thick and smooth; ventral tibial apophysis triangular; retrolateral tibial apophysis rectangular, with an undulated tip, almost uniform in width (Fig. 2J–M), *versus* embolic base very thick and roundly protruding; ventral tibial apophysis falcate; retrolateral tibial apophysis caudate, with a depressed tip, the middle section distinctly narrower than the base and tip. Female – epigynal atrium semi-circular; anterior rim of sclerotised epigynal fold angularly curved; copulatory duct looped and thinner than the receptaculum; receptaculum globular; globular mound indistinct; fertilisation duct almost horizontally arranged (Fig. 2G–I), versus epigynal atrium comma-shaped; anterior rim of sclerotised epigynal fold slightly curved; copulatory duct not looped and almost as thick as the receptaculum; receptaculum elongate-oval; globular mound distinct and gently elevated; fertilisation duct almost vertically arranged in *P. guiyang* (Long et al. (2022): 118, figs. 3A–D; Wang et al. (2024): 281, figs. 1C and D, 2C–E; Zhang et al. (2025): 343, figs. 8C–G, 9A–D). Comparison with *P. subaureolus*: Male – tegulum elliptical; embolic base thick and smooth; ventral tibial apophysis strongly sclerotised; retrolateral tibial apophysis with a gently undulated tip, the middle section flat (Fig. 2J–M), versus tegulum round; embolic base very thick and angularly protruding; ventral tibial apophysis weakly sclerotised; retrolateral tibial apophysis with a sharply undulated tip, the middle section depressed. Female - epigynal atrium wide and semi-circular; anterior rim of sclerotised epigynal fold angularly curved; globular mound indistinct; fertilisation duct almost horizontally arranged versus epigynal atrium narrow and elongate-oval; anterior rim of sclerotised epigynal fold slightly curved; globular mound roundly distinct; fertilisation duct vertically arranged in *P. subaureolus* (Zhang et al. (2025): 343, figs. 10D–F, 11D and E).

#### Etymology

The specific name is a noun in apposition referring to the type locality, Uljin-gun, Gyeongsangbuk-do.

#### Distribution

Korea (Uljin-gun and Yeongyang-gun, Gyeongsanbuk-do) (Fig. 1).

#### Habitat

Shrubby bush layer in a mountainous mixed forest.

## Discussion

Within Northeast Asia, 25 species of *Philodromus* have been recorded from Korea, Japan, China and the Russian Far East, including *P. aliensis*, *P. aureolus*, *P. auricomus*, *P. buxi*, *P. cespitum*, *P. chambaensis*, *P. daoxianen*, *P. digitatus*, *P. fuscomarginatus*, *P. guiyang*, *P. gyirongensis*, *P. lasaensis*, *P. lhasana*, *P. margaritatus*, *P. nigrostriatipes*, *P. paiki*, *P. poecilus*, *P. renarius*, *P. roseus*, *P. rufus*, *P. shaochui*, *P. spinitarsis*, *P. subaureolus*, *P. utotchkini* and *P. vinokurovi* (World Spider Catalog 2025). The discovery and description of *P. uljin* sp. nov. significantly contribute to our understanding of biodiversity within the family Philodromidae in this region. The addition of this new species increases the number of *Philodromus* species recorded in South Korea and highlights the need for continued and intensive faunal surveys, particularly in the country’s relatively understudied mountainous regions. Moreover, this study emphasises that, despite the growing number of taxonomic studies of *Philodromus* in recent years, the genus remains taxonomically challenging and in need of a comprehensive regional or global revision. Although most studies addressing the phylogenetic relationships amongst genera of Philodromidae have focused on Palaearctic taxa, particularly the genus *Philodromus* Walckenaer, 1826 (Muster et al. 2007, Muster 2009), only a very limited number of Northeast Asian species have been examined. To address this limitation, further studies incorporating molecular approaches, including DNA barcoding, are necessary to improve our understanding of the genus and to clarify both regional and global phylogenetic relationships amongst its congeners. In addition, future ecological investigations focusing on distribution, microhabitat preferences, life history and seasonal phenology will provide deeper insights into the evolutionary adaptations of this genus.

## Supplementary Material

XML Treatment for Philodromus uljin
